# A New Perspective on the Cavernous Sinus as Seen through Multiple Surgical Corridors: Anatomical Study Comparing the Transorbital, Endonasal, and Transcranial Routes and the Relative Coterminous Spatial Regions

**DOI:** 10.3390/brainsci13081215

**Published:** 2023-08-17

**Authors:** Sergio Corvino, Pedro L. Villanueva-Solórzano, Martina Offi, Daniele Armocida, Motonobu Nonaka, Giorgio Iaconetta, Felice Esposito, Luigi Maria Cavallo, Matteo de Notaris

**Affiliations:** 1 Division of Neurosurgery, Department of Neuroscience and Reproductive and Odontostomatological Sciences, Università di Napoli “Federico II”, 80131 Naples, Italy; sercorvino@gmail.com (S.C.); lcavallo@unina.it (L.M.C.); 2 PhD Program in Neuroscience, Department of Neuroscience and Reproductive and Odontostomatological Sciences, Università di Napoli “Federico II”, 80131 Naples, Italy; 3 Department of Neurosurgery, National Institute of Neurology and Neurosurgery “Manuel Velasco Suarez”, Mexico City 14269, Mexico; pedrovs@me.com; 4 Institute of Neurosurgery, Fondazione Policlinico Universitario A. Gemelli, 00168 Rome, Italy; martinaoffi.mo@gmail.com; 5 Division of Neurosurgery, Catholic University of Rome, 00153 Rome, Italy; 6 Neurosurgery Division, Human Neurosciences Department, “Sapienza” University, 00185 Rome, Italy; danielearmocida@yahoo.it; 7 Department of Neurosurgery, Kochi University Hospital, 185-1, Oko-cho, Kohasu, Kochi 783-8505, Japan; mtb.nonaka@gmail.com; 8 Neurosurgical Clinic A.O.U. “San Giovanni di Dio e Ruggi d’Aragona”, 84131 Salerno, Italy; iaconetta@libero.it; 9 Neurosurgery Operative Unit, Department of Neuroscience, Coordinator Neuroanatomy Section Italian Society of Neurosurgery, G. Rummo Hospital, 82100 Benevento, Italy; matteodenotaris@gmail.com

**Keywords:** cavernous sinus triangles, endoscopic transorbital, extended endoscopic endonasal, fronto-temporo-orbito-zygomatic, middle fossa

## Abstract

***Background***: The cavernous sinus (CS) is a highly vulnerable anatomical space, mainly due to the neurovascular structures that it contains; therefore, a detailed knowledge of its anatomy is mandatory for surgical unlocking. In this study, we compared the anatomy of this region from different endoscopic and microsurgical operative corridors, further focusing on the corresponding anatomic landmarks encountered along these routes. Furthermore, we tried to define the safe entry zones to this venous space from these three different operative corridors, and to provide indications regarding the optimal approach according to the lesion’s location. ***Methods***: Five embalmed and injected adult cadaveric specimens (10 sides) separately underwent dissection and exposure of the CS via superior eyelid endoscopic transorbital (SETOA), extended endoscopic endonasal transsphenoidal-transethmoidal (EEEA), and microsurgical transcranial fronto-temporo-orbito-zygomatic (FTOZ) approaches. The anatomical landmarks and the content of this venous space were described and compared from these surgical perspectives. ***Results***: The oculomotor triangle can be clearly exposed only by the FTOZ approach. Unlike EEEA, for the exposure of the clinoid triangle content, the anterior clinoid process removal is required for FTOZ and SETOA. The supra- and infratrochlear as well as the anteromedial and anterolateral triangles can be exposed by all three corridors. The most recently introduced SETOA allowed for the exposure of the entire lateral wall of the CS without entering its neurovascular structures and part of the posterior wall; furthermore, thanks to its anteroposterior trajectory, it allowed for the disclosure of the posterior ascending segment of the cavernous ICA with the related sympathetic plexus through the Mullan’s triangle, in a minimally invasive fashion. Through the anterolateral triangle, the transorbital corridor allowed us to expose the lateral 180 degrees of the Vidian nerve and artery in the homonymous canal, the anterolateral aspect of the lacerum segment of the ICA at the transition zone from the petrous horizontal to the ascending posterior cavernous segment, surrounded by the carotid sympathetic plexus, and the medial Meckel’s cave. ***Conclusions***: Different regions of the cavernous sinus are better exposed by different surgical corridors. The relationship of the tumor with cranial nerves in the lateral wall guides the selection of the approach to cavernous sinus lesions. The transorbital endoscopic approach can be considered to be a safe and minimally invasive complementary surgical corridor to the well-established transcranial and endoscopic endonasal routes for the exposure of selected lesions of the cavernous sinus. Nevertheless, peer knowledge of the anatomy and a surgical learning curve are required.

## 1. Introduction

The cavernous sinus (CS) is a vulnerable venous space located deep in the center of the base of the skull, and it includes vital and highly functional neurovascular structures—e.g., the internal carotid artery (ICA) and its branches; the III, IV, and VI cranial nerves; and the ophthalmic division of the trigeminal nerve. Therefore the detailed knowledge of its anatomy is mandatory to avoid potentially life-threating iatrogenic injuries. 

As already happened in the past with the advent of the endoscopic endonasal approaches (mainly addressed to the pathologies of the midline skull base through a ventral median corridor), most recently the endoscopic transorbital route has opened a new window, but mainly for the paramedian and lateral aspects of the anterior and middle cranial fossae up to the petrous apex [1,2]. 

Since the first pioneering work of Parkinson [3], which describes the surgical approach to a carotid-cavernous fistula, several anatomical studies [4,5,6,7,8,9,10] and surgical series [11,12,13,14], have provided a detailed description of this dural envelope and its safe entry zones from different routes, both transcranial [3,15,16,17] and endonasal [16,17,18,19,20], and most recently endoscopic transorbital [21,22,23], each of them with related pro and cons.

Nevertheless, a single approach is not sufficient to expose the entire CS and simultaneously to have the proximal and distal control of the cavernous ICA, which is necessary for a safe surgery. 

Tumoral, congenital, infectious/inflammatory/granulomatous, and vascular pathologies [24], of any size and morphology, pattern of growth, and diffusion, can involve one or more compartments of the cavernous sinus and have different relationships with the structures lying within it. In this scenario, when a surgical treatment is indicated, it is mandatory for a neurosurgeon to be confident with more than one surgical route to ensure the availability of different working angles through the various triangles to maximize the tumor resection and to minimize the risk of injury to the cranial nerves and ICA, ultimately choosing the best tailored option.

**Figure 1:**
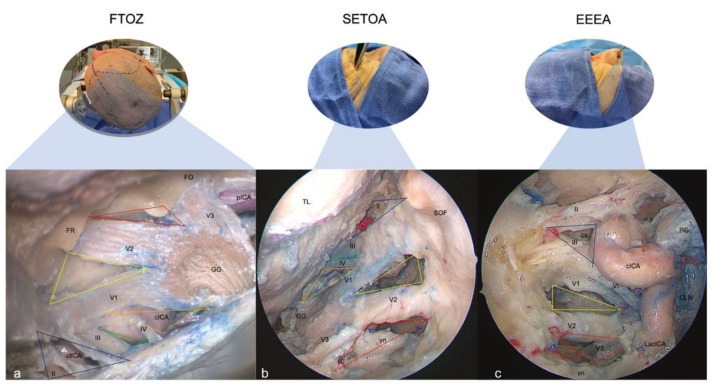
Exposure of the right-side cavernous sinus from different surgical perspectives: (**a**) Fronto-temporo-orbito-zygomatic (FTOZ) approach: head specimen secured in three-pin Mayfield skull clamp, 45 degrees rotated and hyperextended so that the malar eminence was the highest point at the horizon line. (**b**) Superior eyelid endoscopic transorbital approach (SETOA): head specimen in neutral supine position, 10 degrees flexed and 10 degrees rotated to the contralateral side of the operator. (**c**) Extended endoscopic endonasal transsphenoidal transethmoidal approach (EEEA): head specimen in neutral supine position, 10 degrees flexed and slightly rotated to the side of the operator. (GG: Gasserian ganglion; TL: temporal lobe; FR: foramen rotundum; FO: foramen ovale; vn: Vidian nerve; cICA: cavernous internal carotid artery; pICA: petrous internal carotid artery; LacICA: lacerum internal carotid artery; clICA: clinoidal internal carotid artery; os: optic strut; SOF: superior orbital fissure; PG: pituitary gland; CLIV: clivus; red lines: anterolateral triangle; yellow lines: anteromedial triangle; orange lines: infratrochlear triangle; green lines: supratrochlear triangle; blue lines: clinoidal triangle).

The aim of the current study was to provide an anatomical description from a superior eyelid transorbital endoscopic (SETOA) perspective of the cavernous sinus and its relationship with the main neurovascular structures; furthermore, we attempted to highlight the main differences and similarities of the exposed area and its landmarks as seen from the endoscopic endonasal and transcranial routes (Figure 1). Finally, we tried to define the safe entry zones to this venous space from these three different operative corridors (Table 1) and provide indications regarding the optimal approach according to the lesion’s location.

## 2. Materials and Methods

Anatomical dissections were performed at the Laboratory of Skull Base and Micro-neurosurgery of the Weill Cornell Neurosurgical Innovations and Training Center, New York, USA. Five adult cadaveric specimens (10 sides), embalmed and injected with red and blue latex for the arteriosus and venous blood vessels, respectively, were dissected. The fronto-temporo-orbito-zygomatic (FTOZ) approach was performed under microscopic visualization (OPMI, Zeiss, Oberkochen, Germany), whereas the extended endoscopic endonasal transsphenoidal-transethmoidal (EEEA) was performed with a rigid endoscope of 4 mm diameter, 18 cm in length, with 0° and 30° rod lenses as optical devices (Karl Storz, Tuttlingen, Germany); finally, the SETOA, after the initial step under macroscopic visualization, proceeded under endoscopic visualization. The endoscope was connected to a light source (300 W Xenon, Karl Storz) through a fiber-optic cable and to an HD camera (Endovision Telecam SL; Karl Storz). 

We adopted the intracranial classification systems (C1–C7) proposed by Bouthillier et al. [25] and by Labib et al. [26] for the description of the anatomical course of the ICA during FTOZ/SETOA and EEEA, respectively, and the well-known division in triangles of the cavernous sinus [15] was used as a reference to depict the areas of exposure. 

### 2.1. Fronto-Temporo-Orbito-Zygomatic (FTOZ) Approach

The surgical procedure started with a curvilinear skin incision extended from 1 cm anterior to the tragus and below the zygomatic arch, to the contralateral mid-pupillary line, and was followed by a subgaleal interfascial dissection [27] and a retrograde subperiosteal detachment of the temporalis muscle [28]. At this point, a two-piece orbitozygomatic craniotomy according to Zabramski’s [29] technique was performed, followed by the cutting of the meningo-orbital band [30] and the extradural anterior clinoidectomy [31]. Finally, the surgical procedure was completed with the interperiosteal-dural dissection of the lateral wall of the CS and the middle cranial fossa, and with pericavernous maneuvers [32] to expand the optic–carotid and carotid–oculomotor windows. 

### 2.2. Superior Eyelid Transorbital Endoscopic Approach (SETOA)

An SETOA to the petrous apex was performed as previously reported in the literature [33]. As previously reported in the pertinent literature [34], the orbital retraction was set at <10 mm.

A skin incision was placed in a superior eyelid wrinkle; hence, once the orbicularis oculi muscle was identified, the dissection was carried out in depth up to the superior orbital rim and extended laterally up to the frontozygomatic suture (FZS). After cutting the periosteum where it became continuous with the periorbita, the dissection continued, with endoscopic assistance, in a subperiosteum/periorbital plane within the orbit until the lateral margin of the inferior and superior orbital fissures. At this point, once the zygomatic body and the intraorbital part of the greater sphenoid wing (including the sagittal crest [35]) were drilled until the exposure of the temporal pole dura mater, an interperiosteal-dural dissection via the meningo-orbital band (MOB) [21] was performed to unlock the lateral wall of the CS up to the Gasserian ganglion (GG). After cutting the middle meningeal artery (MMA), the temporal pole was elevated in an extradural fashion and, once the mid-subtemporal ridge and the trigeminal lateral loop were identified [36], the anterolateral triangle of middle fossa was opened [37]. Finally, an extradural anterior clinoidectomy [38] completed the surgical procedure.

### 2.3. Extended Endoscopic Endonasal Transsphenoidal Transethmoidal Approach (EEEA)

An extended endoscopic endonasal transsphenoidal transethmoidal approach was performed as previously reported in the literature [20,39]. In contrast to the standard endoscopic endonasal transsphenoidal approach, to obtain a wider exposition of the CS, the sphenoidotomy was extended more laterally and the posterior ethmoidal cells were opened. Furthermore, to expand the operative corridor, the uncinate process was removed and the bulla ethmoidalis was opened, allowing us to reach and remove the anterior ethmoid cells. The removal of the posterior ethmoid cells and the anterior wall of the sphenoid sinus allowed us to expose the lateral wall of the sphenoid sinus with a direct trajectory, and once it was removed, the CS came into view. 

## 3. Results

The differences and likenesses of the various regions of the CS and the related anatomical landmarks were analyzed to make a descriptive comparison from transcranial, endoscopic endonasal, and endoscopic transorbital perspectives.

### 3.1. Clinoid Triangle (Dolenc’s Triangle)

#### 3.1.1. FTOZ Perspective

**Figure 2:**
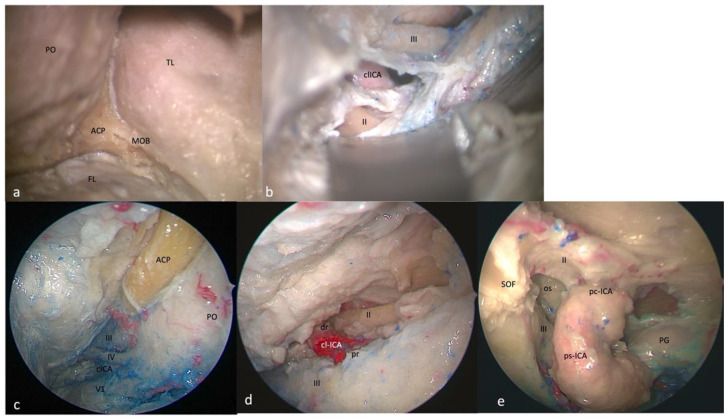
Right-side clinoidal (Dolenc’s) triangle: FTOZ perspective (**a**) before and (**b**) after anterior clinoidal process removal. SETOA perspective (**c**) before and (**d**) after anterior clinoidal process removal. (**e**) EEEA perspective with preserved anterior clinoidal process. The boundaries of this triangles are represented by the inferior margin of the optic nerve superiorly, the superior margin of the oculomotor nerve inferiorly, and by the segment of the anterior petroclinoid dural fold between the entry point of the II and III cranial nerves (PO: periorbit; ACP: anterior clinoid process; TL: temporal lobe; MOB: meningo-orbital band; FL: frontal lobe; cl-ICA: clinoidal internal carotid artery; dr: distal ring; pr: proximal ring; SOF: superior orbital fissure; os: optic strut; ps-ICA: parasellar internal carotid artery; pc-ICA: paraclinoid internal carotid artery; PG: pituitary gland).

This area is bounded by the inferior margin of the optic nerve superiorly, the superior margin of the oculomotor nerve inferiorly, and by the segment of the anterior petroclinoid dural fold between the entry point of the II and III cranial nerves. It includes the anterior clinoid process, which covers the clinoidal segment of the ICA (C5 segment [25]) between the proximal and distal dural rings. It is necessary to remove the anterior clinoidal process to expose its content (Figure 2a,b). 

#### 3.1.2. SETOA Perspective

The clinoid triangle is completely exposed through transorbital route and, after the anterior clinoidectomy, its content is also evident. 

The removal of the anterior clinoid process (ACP) reveals a pyramidal dural pocket bounded by the dura on the superior surface of the lesser sphenoid wing (LSW) laterally, the dura on the superior surface of the LSW and attached to the lateral edge of the planum sphenoidale superiorly, the falciform ligament covering the proximal segment of the optic nerve (ON) after its unroofing medially, and finally, the optic nerve dural sheath and the anterior part of the distal dural ring of the ICA inferiorly.

The ON is observed along its posteromedial course toward the optic chiasm. The clinoidal segment of the ICA (C5 segment [25]) between the lower and upper dural rings is observed in the central part of the triangle, with the ophthalmic artery running inferomedially to the ON. Finally, the optic–carotid membrane and space are also evident (Figure 2c,d).

#### 3.1.3. EEEA Perspective

This area is bounded by the optic nerve above and the oculomotor nerve below the optic strut. The base of this triangle is represented by the distal part of the parasellar and paraclinoid segments of the ICA [26]. The content of this triangle is the optic strut or optic–carotid recess (Figure 2e).

### 3.2. Oculomotor Triangle (Hakuba’s Triangle)

#### 3.2.1. FTOZ Perspective

This triangle forms the posterior part of the roof of the cavernous sinus. Its boundaries are the interclinoid dural fold medially and the anterior and posterior petroclinoid dural folds laterally and posteriorly, respectively.

#### 3.2.2. SETOA Perspective

This area, even after anterior clinoidectomy, is scarcely recognizable from this surgical corridor.

#### 3.2.3. EEEA Perspective

This triangle is also not identifiable after gentle medial displacement of the ICA.

### 3.3. Supratrochlear Triangle (Paramedian)

#### 3.3.1. FTOZ Perspective

**Figure 3:**
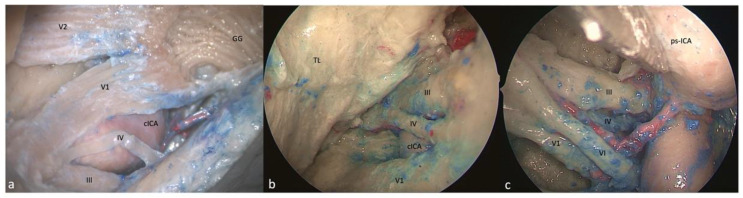
Right-side supra- and infratrochlear triangles: (**a**) FTOZ, (**b**) SETOA, and (**c**) EEEA perspectives. The boundaries of the supratrochlear are represented by the inferior border of the III c.n. superiorly, the superior border of the IV c.n. inferiorly, and the segment of the dura of the roof of the cavernous sinus between the entry points of these two nerves; regarding the infratrochlear triangle, it is delimited superiorly by the lower margin of the trochlear nerve, inferiorly by the upper margin of V1, and posteriorly by the line connecting the point where the trochlear nerve enters the roof of the cavernous sinus and the point where the trigeminal nerve enters the Meckel’s cave (GG: Gasserian ganglion; cICA: cavernous internal carotid artery; TL: temporal lobe, ps-ICA: parasellar internal carotid artery).

This area is bounded by the inferior border of the oculomotor nerve superiorly, the superior border of the trochlear nerve inferiorly, and the segment of the dura of the roof of the cavernous sinus between the entry points of these two nerves. Its content is represented by the horizontal cavernous ICA (Figure 3a).

#### 3.3.2. SETOA Perspective

This triangle and its boundaries are completely exposed via the transorbital corridor just after interperiosteal-dural dissection of the lateral wall of the CS. This is a very narrow space that medially hides the horizontal segment of the cavernous ICA (C4 segment [25]) (Figure 3b).

#### 3.3.3. EEEA Perspective

Only the apex of this triangle, where the III and IV cranial nerves converge toward the SOF, can be exposed, and the medial displacement of the ICA and/or the lateral displacement of the cranial nerves is required (Figure 3c). 

### 3.4. Infratrochlear Triangle (Parkinson’s triangle)

#### 3.4.1. FTOZ Perspective

This triangle is delimited superiorly by the lower margin of the trochlear nerve, inferiorly by the upper margin of V1, and posteriorly by the line connecting the point where the trochlear nerve enters the roof of cavernous sinus and the point where the trigeminal nerve enters the Meckel’s cave. This region hosts the posterior bend of the cavernous ICA (C4 segment [25]) with its branches (meningohypophyseal trunk) (Figure 3a).

#### 3.4.2. SETOA Perspective

This region and its content are completely exposed via the transorbital route; just after interperiosteal-dural dissection of the lateral wall of the CS, the anterolateral aspect of the superior half of the ascending segment of the cavernous ICA is evident. Furthermore, after a gentle downward retraction of the proximal part of V1, it is possible to identify the sixth cranial neve exiting from the Dorello’s canal under the Gruber’s ligament and coursing anteriorly in the lateral wall of the CS medially to V1. After a gentle upward displacement of the trochlear nerve, it is possible to expose the meningohypophyseal trunk (MHT) that arises from the posterior bend of cavernous ICA (C4 segment [25]) (Figure 3b). 

#### 3.4.3. EEEA Perspective

Only the anterior narrow space of this triangle and its content, represented by the inferolateral trunk of the cavernous ICA, are evident, because of the abducens nerve that covers the ophthalmic division of the trigeminal nerve, and because of the parasellar ICA [26] that obstructs access to the posterior compartment (Figure 3c). 

### 3.5. Anteromedial Triangle (Mullan’s Triangle)

#### 3.5.1. FTOZ Perspective

**Figure 4:**
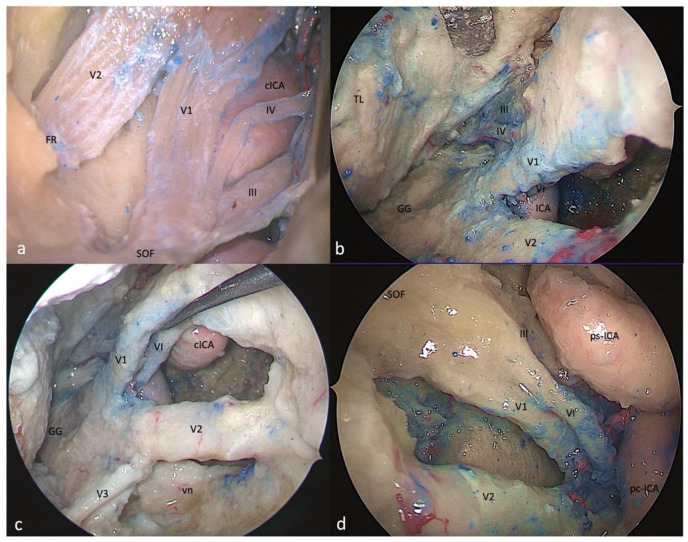
Right-side anteromedial (Mullan’s) triangle: (**a**) FTOZ, (**b**,**c**) SETOA, and (**d**) EEEA perspectives. This region is delimited superiorly by the lower margin of V1, inferiorly by the upper margin of V2, and anteriorly by the line connecting the point where the ophthalmic nerve enters the superior orbital fissure and the point where the maxillary nerve enters the foramen rotundum (FR: foramen rotundum; SOF: superior orbital fissure; cICA: cavernous internal carotid artery; TL: temporal lobe; GG: Gasserian ganglion; vn: Vidian nerve; ps-ICA: parasellar internal carotid artery; pc-ICA: paraclival internal carotid artery).

This region is delimited superiorly by the lower margin of V1, inferiorly by the upper margin of V2, and anteriorly by the line connecting the point where the ophthalmic nerve enters the superior orbital fissure and the point where the maxillary nerve enters the foramen rotundum (Figure 4a). The removal of the outer bony shell of this triangle leads into the sphenoid sinus.

#### 3.5.2. SETOA Perspective

This region comes immediately into the endoscopic view after interperiosteal-dural dissection of the lateral wall of the CS, and it is the largest safe entry zone to the CS. Unlike the transcranial frontotemporal point of view, where no segments of the ICA are visible in this area, the SETOA allows for the in-depth disclosure, at the apex of this triangle, where V1 and V2 converge, of the inferior half of the posterior ascending segment of the cavernous ICA (C4 segment [25]), surrounded by sympathetic fibers of the carotid plexus, passing medially to the petro-lingual ligament to reach the cavernous sinus (Figure 4b). In this triangle, the VI cranial nerve courses almost horizontally, medially to V1 and laterally to the ICA, towards the SOF, and it can be visualized after gentle upward retraction of V1 (Figure 4c). One fundamental landmark that guides surgical dissection of the anteromedial triangle, avoiding entering the CS space, is the foramen rotundum, which is encountered after resection of the sagittal crest that discloses V2 inferiorly and outside the CS. Surgical dissection must be performed between the two trigeminal branches at the level of the superior edge of V2, dissecting the perineurium covering the two nerves in an anteroposterior direction to free the two branches, and mobilizing the ophthalmic nerve superiorly to expand the space between them and gain access to the posteroinferior portion of the CS.

#### 3.5.3. EEEA Perspective

This area and its content, represented by venous structures, are completely exposed after bone removal from the lateral wall of the sphenoid sinus, with V1 partially hidden by the sixth cranial nerve. The apex of this triangle, where V1 and V2 converge, can be disclosed after medial displacement of the paraclival and parasellar segments of the ICA [26] (Figure 4d). 

### 3.6. Anterolateral Triangle 

#### 3.6.1. FTOZ Perspective

**Figure 5:**
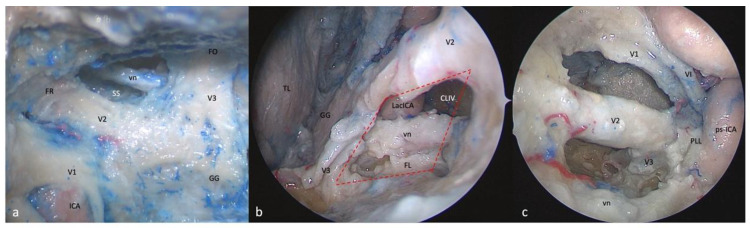
Right-side anterolateral triangle: (**a**) FTOZ, (**b**) SETOA, and (**c**) EEEA perspectives. This area is bounded by the lower border of V2 superiorly, the upper border of V3, inferiorly, and the line that connects the foramina rotundum and ovale (red dotted lines: quadrangular space; FO: foramen ovale; FR: foramen rotundum; GG: Gasserian ganglion; CLIV: clivus; Lac ICA: lacerum internal carotid artery; vn: Vidian nerve; FL: foramen lacerum; MC: Meckel’s cave; cICA: cavernous internal carotid artery; ps-ICA: parasellar internal carotid artery).

This area is bounded by the lower border of V2 superiorly, the upper border of V3 inferiorly, and the line that connects the foramina rotundum and ovale. The drilling of its medial wall exposes the sphenoid sinus (Figure 5a). 

#### 3.6.2. SETOA Perspective

The opening of this triangle allows for the disclosure of the Vidian nerve and artery in the homonymous canal along their course up to the anterolateral edge of the foramen lacerum, where the posterior opening of the canal is filled with cartilaginous tissue that blends into the more medially positioned cartilage filling the foramen lacerum. The lacerum segment of the ICA, at its transition zone from the horizontal petrous segment to the ascending cavernous segment, located medially to the petrolingual ligament, along with the related carotid sympathetic plexus, can be also exposed (Figure 5b). As recently already described by our group [37], it is possible to appreciate a space limited by the inferior border of V2 superiorly, the superior border of V3 posteriorly, the line crossing the most anterior limit of exposure of the Vidian nerve and joining the foramen rotundum and the point where the greater wing joints the body of the sphenoid bone anteriorly, and the line between this last point and the foramen ovale posteriorly (red dotted line). This area includes two windows divided by the course of the Vidian nerve until where it blends into the cartilaginous tissue of the FL under the trigeminal nerve, and which unfold different corridors:

(a) A wider superior window (“supravidian”) that discloses two corridors in relationship to the lacerum segments of the ICA: a “medial supravidian corridor” leading to the lower clivus, and a “lateral supravidian corridor” leading, after gentle lateralization of the Gasserian ganglion, to the medial aspect of the Meckel’s cave and the terminal portion of the horizontal petrous ICA (pICA).
(b) A narrow inferior window (“infravidian”) that includes the inferior portion of the foramen lacerum distally, and the sphenoid sinus proximally.

#### 3.6.3. EEEA Perspective

Whereas V2 is disclosed from the origin up to the foramen rotundum, V3 is recognizable only in its course from the origin at the Gasserian ganglion up to the entrance the foramen ovale (Figure 5c). 

## 4. Discussion

### 4.1. Anatomical Considerations

The oculomotor triangle can be clearly exposed and expanded by opening the optic–carotid and carotid–oculomotor windows only through the transcranial approach, whereas it is scarcely identifiable from SETOA and EEEA in normal conditions; however, Nunes et al. [40]. have described an endoscopic endonasal transoculomotor triangle approach for adenomas invading the parapeduncolar space in which the lesion created the corridor. 

To expose the clinoidal triangle and its content, anterior clinoidectomy is necessary for both SETOA and FTOZ. Unlike the transcranial route, extradural clinoidectomy via the transorbital route does not have anatomical landmarks, and the limits of the ACP base drive the drilling [38]. Unlike the transcranial and transorbital approaches, during EEEA the ICA comes into the view in front of the ACP, just after exposing the posterior wall of the sphenoid sinus. We consider the access to this area to be safe from all three operative corridors (“low risk”, green traffic light—Table 1). 

The transorbital corridor provides the same exposure of the supra- and infratrochlear triangles’ contents as the transcranial route, but with a different working angle, and provides a better control on the anterior aspect of the posterior ascending segment of the cavernous ICA. The EEEA allows for the exposure of just the apex of the supratrochlear triangle, but the medial displacement of the ICA and/or the lateral displacement of cranial nerves are required; concerning the Parkinson’s triangle, the horizontal cavernous ICA and the VI nerve partially obstruct its exposure. We consider the access to both of these triangles to be relatively safe from all three corridors (“mid risk”, yellow traffic light—Table 1), taking into the account the superficial course of the ICA and MHT in this region from each of the different perspectives. 

The anteromedial triangle is the largest window opened on the CS from SETOA. Unlike the transcranial route, through this route, the endoscopic transorbital route allows for the exposure of the anterior aspect of the inferior half of the posterior ascending segment of the cavernous ICA, surrounded by sympathetic fibers of the carotid plexus; additionally, the VI cranial nerve, along its almost horizontal course (medially to V1 and laterally to the ICA, towards the SOF), can be visualized after gentle upward retraction of V1. We consider the access to this area from EEEA and FTOZ to be safe (“low risk”, green traffic light—Table 1), whereas one should be more careful when using the transorbital corridor (“mid risk”, yellow traffic light—Table 1) due to the proximity of the inferior border of the horizontal segment of the cavernous ICA, partially hidden by V2 and the inferior segment of the posterior ascending cavernous ICA deep in the triangle. 

**Figure 6:**
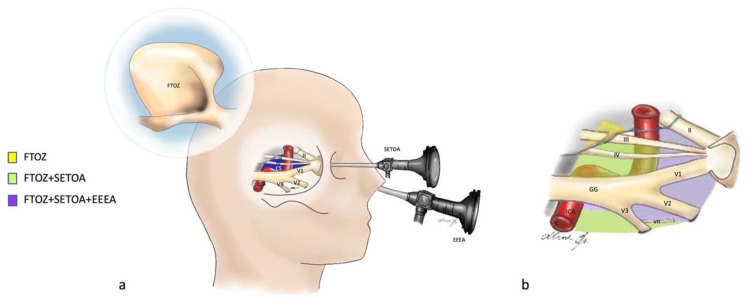
(**a**) Graphic showing the fronto-temporo-orbito-zygomatic (FTOZ), endoscopic transorbital (SETOA) and endoscopic endonasal (EEEA) approaches to the cavernous sinus; CS: cavernous sinus; vn: Vidian nerve; GG: Gasserian ganglion; blue = cavernous sinus; yellow: cranial nerves; red: ICA. (**b**) Graphic of the cavernous sinus and its exposure areas from the three approaches through the different triangles: yellow space: this area can be exposed only through FTOZ (oculomotor triangle); green space: these areas can be separately exposed only through FTOZ and SETOA (supra- and infratrochlear triangles, anterolateral triangle laterally to the Vidian nerve, and posteromedial and posterolateral triangles of the middle fossa, i.e., Glassock’s and Kawase’s triangles); purple space: these areas can be separately exposed through FTOZ, SETOA, and EEEA (clinoid, supra- and infratrochlear, and anteromedial and anterolateral triangles).

The opening of the anterolateral triangle through SETOA [37] reveals a space that can be divided into a wider superior window (“supravidian”) and a narrow inferior window (“infravidian”). The supravidian window allows direct access to the lacerum segment of the ICA and the related carotid sympathetic plexus; furthermore, this space reveals two different corridors: the *medial supravidian corridor* leading to the lower clivus, and the *lateral supravidian corridor* leading to the Meckel’s cave and the terminal portion of the horizontal petrous ICA, medial and lateral to the lacerum ICA, respectively. We consider the access to this area to be safe from all three (FTOZ, SETOA, and EEEA) surgical routes (“low risk”, green traffic light—Table 1) (Figure 6).

### 4.2. Surgical Nuances

Among the more or less recent classifications of the compartments of the CS [4,41,42], in agreement with that provided by Harris et al. [4], we considered three main venous spaces in relation with the course of the cavernous ICA: posterosuperior, anteroinferior, and medial compartments.

The FTOZ route allows access to the CS through its roof and lateral wall using the clinoidal/oculomotor and the supra/infratrochlear triangles, respectively [43].

The clinoidal triangle represents the lower floor of the anterior portion of the roof of the cavernous sinus, and it is commonly used to approach paraclinoid or carotid–ophthalmic aneurysms; its exposure requires the intra- or extradural removal of the ACP, and care must be taken regarding the clinoidal ICA and the unroofing of the optic canal during this maneuver. The exposure of this area continues with the opening of the optic sheath and the distal dural ring. The oculomotor triangle represents the posterior part of the roof of the cavernous sinus, and it is usually used as corridor to access basilar tip aneurysms and tumors inside the cavernous sinus; its exposure requires the opening of the oculomotor cistern, the incision of the carotid–oculomotor membrane, and the incision of the dura of the triangle; care must be taken to identify the MHT. 

The simultaneous opening of these two triangles allows for the management of lesions involving the lateral, posterosuperior, and medial compartments of the CS. 

The FTOZ allows access to the posterosuperior and anteroinferior compartments of the CS, as well as through its lateral wall by opening the supra- and/or infratrochlear triangles, just after the peeling of the middle fossa; care is recommended to identify the VI c.n. exiting the Dorello’s canal in the Parkinson’s triangle. 

The EEEA allows access to the posterosuperior and anterior compartments of the CS through its medial (sellar) and anterior (sphenoidal) walls thanks to medial-to-lateral [44] and anterior-to-posterior trajectories [41], respectively. To access the posterior compartment, the opening of the sellar dura and the cutting of the inferior hypophyseal artery are required; access to the anteroinferior compartment is facilitated by using a transpterygoid approach. 

The exposure area of the lateral wall of the CS via EEEA is greatly influenced by the ICA’s position: because of its localization inside the CS, the ICA comes into the endoscopic view before the lateral wall of the CS, representing an obstacle for the visualization of the posterior content of the CS’s lateral wall—mainly the supra- and infratrochlear triangles. Conversely, the anterior part of the clinoidal triangle, the entire anteromedial triangle, and the upper part of the anterolateral triangle are exposed and provide potential pathways from this route to the middle cranial fossa. 

As this route is mainly indicated for midline skull-base pathologies, its main risks and drawbacks in approaching the CS are related to the lateral extension, i.e., pituitary adenomas with CS invasion, and include the injury of the ICA and the cranial nerves, CSF leakage, and limited resection rates. 

The interperiosteal-dural dissection from anterior to posterior via the MOB [21] during SETOA provides a shorter and direct route, and it follows a natural sagittal plane, allowing for the exposure of the entire lateral wall of the CS without violating its neurovascular compartment, and with an optimal angle of attack [21]. The MOB represents the key landmark for identifying the CS via SETOA. If from one side the extradural endoscopic transsphenoidal and transethmoidal approaches offer direct access to the anterior portion of the cavernous sinus, from the other side the lateral and posterior walls instead represent a challenge from this route [20,45,46]. 

The transorbital approach respects the principles of the modern, minimally invasive skull-base techniques: flattening the skull base and using the extradural space to approach the target lesion, reducing the brain retraction [47]. Nevertheless, a mandatory consideration must be kept in mind when exposing the CS: the width of the surgical corridor. This route, while providing a wide visualization, uses a narrow (and single if compared to EEEA) surgical corridor, limited by orbital rims superiorly, laterally, and inferiorly, and by the vulnerable orbital content medially, which imposes limitations on the surgical freedom and working angles; therefore, it is mainly suitable for small lesions involving the CS at its posterosuperior and anteroinferior lateral compartments, through the supra/infratrochlear and anteromedial/anterolateral triangles, respectively [45]. Peri- and postoperative complications related to the intraoperative orbital content retraction may be avoided with careful management of the globe through the use of a corneal protector, placing the malleable ribbon retractor tangentially against the globe, while constantly monitoring the pupillary diameter during the procedure and removing the instruments from the surgical field every 20–30 min to minimize the pressure on the globe [34].

The relationship of lesion and cranial nerves in the lateral wall, the anatomical course of the ICA, and the displacement of the ICA by the pathology guide the choice of the approach [45]: lesions that displace the cranial nerves laterally are more suitable for EEEA; lesions that displace the cranial nerves medially are more suitable for FTOZ and SETOA. 

The most serious and potentially life-threatening complication is represented by the iatrogenic injury of the ICA, the incidence of which ranges from 3% to 8% [48] in conventional open approaches and is less than 1% during EEEA [49]; no data are reported about the transorbital route for the recent adoption of this technique in the neurosurgical field; therefore, unnecessary exposure of the ICA must be avoided. 

### 4.3. Limitations of This Study

Pure anatomical studies have the common limitation related to cadaveric specimens: the property of cadaveric tissue considerably differs from real anatomy, e.g., variability in size and pneumatization of the sphenoid sinus; the trajectory of the internal carotid artery and cranial nerves; bony protuberances of the skull base. However, the main anatomical relationships between the cavernous sinus and its neurovascular structures are valid and reliable, albeit there is a lack of quantitative analysis. 

## 5. Conclusions

The three operative corridors investigated provide three different points of view of the same anatomical region; each of them has its pros and cons; some areas of the cavernous sinus are better exposed from different approaches.

In this scenario, the transorbital endoscopic approach can be considered to be a safe, complementary route to the well-established transcranial and endoscopic endonasal ones for exploring the cavernous sinus. Nevertheless, as with any new technique, it requires a learning curve, and further clinical series are expected to validate these findings. 

## Figures and Tables

**Table 1:** Summary of the anatomical and surgical aspects of the cavernous sinus triangles from superior eyelid endoscopic transorbital, fronto-temporo-orbito-zygomatic, and extended endoscopic endonasal transsphenoidal-transethmoidal approaches.

Triangles	Clinoidal *(Dolenc)*			Supratrochlear			Infratrochlear *(Parkinson)*			Anteromedial *(Mullan)*			Anterolateral		
**Boundaries**	- Lower border of II c.n. superiorly; - Upper border of III c.n. inferiorly			- Lower border of III c.n. superiorly; - Upper border of IV c.n. inferiorly			- Lower border of IV c.n. superiorly; - Upper border of V1 inferiorly			- Lower border of V1 superiorly; - Upper border of V2 inferiorly			- Lower border of V2 superiorly; - Upper border of V3 inferiorly		
**Approach**	FTOZ	SETOA	EEEA	FTOZ	SETOA	EEEA	FTOZ	SETOA	EEEA	FTOZ	SETOA	EEEA	FTOZ	SETOA	EEEA
**Surgical maneuvers for the exposition**	Anterior clinoidectomy (augmented by peri-cavernous maneuvers)	Anterior clinoidectomy	---	Peeling of the LWCS and middle fossa	Peeling of the LWCS and middle fossa	---	Peeling of the LWCS and middle fossa	Peeling of the LWCS and middle fossa	---	Peeling of the LWCS and middle fossa	Peeling of the LWCS and middle fossa	Removal of lateral wall of the sphenoid sinus	Peeling of the LWCS and middle fossa	Peeling of the LWCS and middle fossa	Removal of lateral wall of the sphenoid sinus
**Content**	cl-ICA, between upper and lower dural rings	cl-ICA, between upper and lower dural rings	Optic strut or optic-carotid recess	hc-ICA and MHT, ILT	hc-ICA	---	Posterior bend of the c-ICA with MHT, VI c.n.	Superior half and posterior bend of posterior c-ICA with MHT	ILT; VI c.n.	Sphenoid sinus	Inferior half of the posterior segment of c-ICA; VI c.n.	Venous structures	Sphenoid sinus	Lacerum ICA; Sympathetic carotid plexus; FL; PLL; Vidian nerve and artery;	---
**Indications**	Meningioma ACP, carotid-ophthalmic aneurysm	Meningioma ACP	---	Schwan-nomas, CS tumors	Schwan-nomas, CS tumors	Pit- adenoma, chondro-sarcomas with lateral extension up to the CS lateral wall	Aneurysm or fistula of proximal c-ICA, CS tumors	Schwannomas, CS tumors	Pit- adenoma, chondro-sarcomas with lateral extension up to the CS lateral wall	Middle fossa tumors with CS invasion	Small lesion involving the anteroinferior compartment of CS	Pit- adenoma, chondro-sarcomas with lateral extension up to the CS lateral wall	---	Small lesion involving the anteroinferior compartment of CS	Pit- adenoma, chondro-sarcomas with lateral extension up to the CS lateral wall
**Safety of surgical access**	Low risk 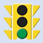	Low risk 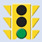	Low risk 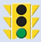	Mid risk 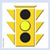	Mid risk 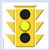	Mid risk 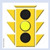	Mid risk 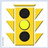	Mid risk 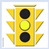	Mid risk 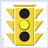	Low risk 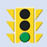	Mid risk 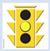	Low risk 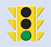	Low risk 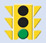	Low risk 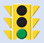	Low risk 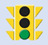

c.n.: cranial nerve; FTOZ: fronto-temporal-orbito-zygomatyc; SETOA: superior eyelid transorbital endoscopic approach; EEEA: extended endoscopic endonasal approach; cl: clinoid; hc: horizontal cavernous; CS: cavernous sinus; c-ICA: cavernous internal carotid artery; Pit: pituitary; LWCS: lateral wall cavernous sinus; ICA: internal carotid artery; PLL: petro-lingual ligament, MHT: meningo-hypophyseal trunk; ILT: inferolateral trunk, FL: foramen lacerum.

## Data Availability

Data used for the current original research are available from the corresponding author upon reasonable request.

## Abbreviations

SETOA	Superior eyelid transorbital endoscopic approach
EEEA	Extended endoscopic endonasal approach
ICA	Internal carotid artery
GG	Gasserian ganglion
MOB	Meningo-orbital band
MMA	Middle meningeal artery
CS	Cavernous sinus
FL	Foramen lacerum
PL	Petrolingual ligament
vn	Vidian nerve
pICA	Petrous internal carotid artery
FTOZ	Fronto-t’emporo-orbito-zygomatic
MC	Meckel’s cave
